# Effect of polyoxyethylene and polyoxypropylene nonionic block copolymers on performance and recruitment of immune cell subsets in weaned pigs

**DOI:** 10.1186/1751-0147-55-54

**Published:** 2013-07-18

**Authors:** Hrvoje Valpotić, Gordan Mršić, Branka Gršković, Daniel Špoljarić, Dubravko Kezić, Siniša Srečec, Mirjana Mataušić-Pišl, Gordana Lacković, Darko Capak, Damir Mihelić, Ksenija Vlahović, Ivica Valpotić, Ahmed Pirkić, Deny Andjelinovic, Maja Popović

**Affiliations:** 1 Veterinary Faculty, University of Zagreb, Heinzelova 55, Zagreb 10 000, Croatia; 2 Forensic Science Center “Ivan Vučetić”, Ilica 335, Zagreb 10 000, Croatia; 3 Križevci College of Agriculture, Mislava Demerca 1, Križevci 48 260, Croatia; 4 Institute for Medical Research, Ksaverska cesta 2, Zagreb 10 000, Croatia; 5 Faculty of Science, University of Zagreb, Rooseveltov trg 6, Zagreb 10 000, Croatia; 6 55 Clinical Hospital Center, Ljubičeva 37, Split 23 000, Croatia

**Keywords:** POE-POP, Weaned pigs, Performance, Immunity

## Abstract

**Background:**

Because European-wide directives are restricting the non-clinical use of antibiotics as in-feed growth promotors in swine production, there is an intensive search for alternative strategies for control and prevention of losses among young pigs. With the growing knowledge of the porcine immune system and its endogenous modulation, it has been clearly established that exogenous immunomodulation using adjuvants and immune response modifiers (IRMs) represents an important prophylactic/therapeutic approach in the prevention/treatment of both stress- and microbial-induced disorders that accompaning weaning. However, it is essential to select a fully evaluated agent which may act either as a nonspecific IRM or synergistically as an adjuvant with vaccines. The synthetic macromolecules with a long history as adjuvant and IRM are nonionic block copolymers which consist of polyoxyethylene (POE) and polyoxypropylene (POP) molecules.

**Methods:**

The aim of this work was to evaluate the effectiveness of POE-POP given as a single peroral dose on productivity parameters such as body weight gain, feed intake and feed conversion ratio, and systemic and intestinal immune parameters by assessing the proportions of CD45^+^ lymphoid cells, CD4^+^ and CD8^+^ T cells, and CD21^+^ B cells in the peripheral blood as well as the number of CD45RA^+^ naive lymphoid cells residing in the ileal mucosa in weaned pigs during a follow-up study 5 weeks after the treatment.

**Results:**

Pigs treated with POE-POP had better feed intake (+ 14.57%), higher average body mass at the end of the experiment (20.91 kg *vs*. 17.61 kg), and higher body weight gain in relation to Day 0 (191.63% *vs*. 144.58%) as well as in relation to nontreated pigs (+ 18.74%), with a lower feed conversion ratio (− 30.26%) in comparison to the control pigs. A much lower diarrhea severity score (5 *vs*. 54) was recorded in pigs treated with POE-POP (− 90.74%) than in the control pigs. A higher average diarrhea severity (ADS) was recorded in the control pigs (1.54 *vs*. 0.14), whereas the treatmant group had much a lower ADS ratio (− 90.91%) after 35 days of the experiment. The pigs that were treated with POE-POP had an increased proportion of CD45^+^, CD4^+^ and CD8^+^ cells at Day 21 (at p < 0.05, p < 0.05 or p < 0.01, respectively), Day 28 (at p < 0.01, respectively) and Day 35 (at p < 0.01, p < 0.05 or p < 0.01, respectively) as well as of CD21^+^ cells at Day 28 (p < 0.05) and Day 35 of the experiment (p < 0.01). Also, these pigs had more numerous CD45RA^+^ cells in interfollicular (p < 0.05) and follicular areas (p < 0.01) of the ileal Peyer’s patches than did control pigs.

**Conclusion:**

This property of POE-POP to induce recruitment of circulating and intestinal immune cell subsets in weaned pigs may allow the use of IRM-active block copolymers as adjuvants for vaccines, particularly those orally delivered and targeted to the gut-associated lymphoid tissues that are well known to promote rather tolerogenic than protective immune responses.

## Background

Numerous intriguing reports have appeared suggesting that a vast variety of substances, whether of synthetic [1] or natural [2] origin can restore or stimulate nonspecific and specific immunity in domestic food animals, particularly in swine and, hence, act as immune response modifiers (IRMs) and/or adjuvants. The synthetic macromolecules with a long history as adjuvant and IRM are poloxameres and poloxamines belonging to a family of more than 50 different amphiphilic nonionic block polymers which consist (poloxameres) of a central hydrophobic polyoxypropylene (POP) molecule, with two hydrophilic chains of polyoxyethylene (POE) or of a slightly different structure (poloxamines), which are tetrafunctional block copolymers with four POE-POP blocks joined by a central ethylene diamine bridge [3]. There were first introduced as surfactants by BASF Co. (Mount Olive, NJ, USA) in the 1950s and since then have found a wide range of potentials in the pharmaceutical and biomedical fields, particularly as adjuvants [4] and IRMs capable of enhancing both humoral and cellular immunity [5,6] by either stimulating antibody formation of protective isotype or modulating inflammation due to their structure and formulation [7]. When applied *per os*, copolymers acted specifically against gut microbiota by forming hydrophilic surfaces at the intestinal mucosa capable of retaining protein molecules and making them more accessible to the antibodies and complement [8]. They were also shown to be potent adjuvants for stimulation of the antibody response against haptenated liposomes in mice [9]. Moreover, they stimulated long-lasting antibody response, increased their titers and may have changed antibody isotype [10].

Both high molecular weight surfactants exhibited either immunomodulatory properties such as phagocyte activation by stimulation of phagocytosis, superoxide anion production and neutrophil degranulation or drug/vaccine delivery to regional lymph nodes as lymphotropic nanopartcles [11,12]. The adjuvant activity following parenteral immunization was thought to arise from the adherence of surfactants to lipids, promoting the retention of the protein antigen in local tissue and facilitating the uptake of the antigen by macrophages [10]. Copolymers with 10% POE preferentially stimulate Type 2 helper T-lymphocyte responses, which support antibody production, including mucosal antibody responses.

Further data showed that nonionic triblock copolymers enhanced presentation of soluble ovalbumin (OVA) to the major histocompatibility complex (MHC) class II-restricted CD4^+^ T cells and MHC class I-restricted CD8^+^ T cells, respectively. Presentation of OVA *via* the class I pathway was enhanced by copolymers in both phagocytic and nonphagocytic antigen presenting cells (APC) [13]. Copolymers with < 10% POE stimulate both Type 1 and Type 2 responses, which support cellular immmune responses and a broader range of humoral immune responses [14]. This property may allow for vaccines to be modulated by using adjuvant-active copolymers that will enhance the most appropriate types of immune responses [15]. A solid adjuvanticity has been shown with parenteral vaccines [16] and with live attenuated oral vaccine against porcine colibacillosis induced by F4ac^+^ enterotoxigenic *E*. *coli* (ETEC) strains [17]. The latter combination showed synergistc effects on CD4a^+^ and CD8a^+^ T cells, CD1^+^ and CD21^+^ B cells, and SWC5^+^ NK cells from the gut-associated lymphoid tissues (GALT) of weaned pigs. The cellular immune response to plasmid DNA vaccines was enhanced by microparticle adjuvant formulation containing nonionic block copolymers in the rhesus monkey [18]. Such copolymers could be also used as nanocarriers for controlled drug delivery and release and/or site-specific targeting [19]. The production of specific anti-F4ac secretory IgA antibodies was increased in weaned pigs primed with POE-POP before the immunization with vaccine candidate F4ac^+^ non-ETEC strain [20]. However, before the use of these copolymers synthesized using various amonts of POE and POP and with different arrangements of their blocks in animals and humans, their adjuvaticity and differing effects on the immune response have to be fully evaluated [21].

The other biological effects induced by nonionic block copolymers of POE-POP include the increase in daily weight gain and overall growth period and morphologic changes in the size of uterus and adrenal glands of an animal, hence, exhibiting the effects similar to those produced by stomatostatin and ACTH [22]. Also, the POE-POP may induce a noncytolytic degranulation of human and murine mast cells with subsequent release of histamine [23,24], and may provide the adjuvant activity by stimulation of transmembrane transport of ions into the cell [25]. More recently, the copolymer when given perorally to weaned pigs increased proportion of their neutrophils and lymphocytes, and the level of glucose while decreasing CRP and haptoglobin levels at days 21, 35 and 7, respectively, following the treatment [26]. Early weaning of pigs is often accompanied by severe diarrhea and growth retardation due to great change in the magnitude and variety of exposure to environmental antigens, loss of immunoregulatory and immunoprotective components of maternal milk and functional immaturity of the immune system, particularly of the GALT, that are not fully developed to the point of actively making either protective responses against harmful microbial antigens or tolerogenic responses to harmless diet components [27]. To help newly weaned pigs to cope with this transition, various nutritional approachesd have been proposed [28], including supplementation of the diet with substances that have properties of anti-microbials and/or IRMs [2].

Thus, the aim of this study was to validate the effectiveness of POE-POP on productivity and performance, stimulation of systemic and local (intestinal) cellular immunity, and maintenance of gut health in weaned pigs based on a follow-up study during 5 weeks after weaning. The influence of treatment was investigated by production parameters such as body weight gain, feed intake and feed conversion ratio and immunological parameters which include: (i) identification and quantification of CD45^+^ lymphoid cells as well as of T (CD4^+^ and CD8^+^) and B (CD21^+^) cells in the peripheral blood by flow cytometry, (ii) localization and distribution of CD45RA^+^ naive lymphoid cells within the ileal mucosa by immunohistology, and (iii) establishing their numerical values by histomorphometry using quantitative image analysis.

## Methods

### Animals

Forty crossbred pigs (Topigs®) of both sexes (females and castrates) with body weight of approximately 6.5 kg, the progeny of four litters (from 3rd parity sows) from a commercial swine farm in eastern Croatia were used. The pigs were weaned at 26 days of age, housed, managed and fed with a standard weaner diet (without antimicrobials or growth promotors) according to rearing technology of the farm. Experimental and animal management procedures were conducted in accordance with the “Directive for the Protection of Vertebrate Animals used for Experimental and other Purposes” (86/609/EEC).

### Study design and procedures

The pigs were randomly divided into two groups comprising 20 animals each, ear-tagged with numbers 1–20 and kept in the same rearing facility of the commercial farm in the separate pens (20 animals in each) that were located in near proximity to each other so the infection might easily spread from one pen to another. Microclimatic conditions and the level of microorganisms in the air were also monitored and although they are not presented in this paper they were within acceptable levels for this phase of swine production. Accordingly, the experimental pigs were kept under the same environmental conditions, and thus, both groups of animals had similar potential for exposure to infectious agents present in this environment during the experimental period. As the animals were of the same breed, weighed approximately 6.5 kg at the weaning age of 26 days, they were considered dependent samples. After two days of acclimation at 28 days of age or Day 0 of the experiment, the pigs were treated as follows: (1) control pigs perorally (*p*.*o*.) received 10 mL of saline, (2) the treatment group *p*.*o*. treated with a single dose of 10 mL with 2.5 mg/mL of POE-POP copolymer preparation (in an oil-in-water emulsion consisting of Drakeol and PBS with 0.5 mg/mL of bovine serum albumin) under formal protect patented BASF name Reverse Tetronic Polyol T150R1 known to be effective as an immunoendocrine modulator in mice (USA patent no. 5.234.683/1993). The preparation also known as Polyphore 32:5 (CytRx, Atlanta, GA, USA) was kindly donated by William L. Ragland from Institute “Ruđer Bošković”, Zagreb, Croatia. Each of the four chains of POE has an average of 5 ethylene oxide moieties while each of the POP chains has an average of 32 moieties. The T150R1 is an 8000 Daltons copolymer such that the portion of the total molecular weight represented by POE constitutes approximately 10% of the compound by weight and the POP portion of the octablock copolymer constitutes approximately 90% of the compound by weight. The experiment was conducted throughout a period of 35 days, the pigs were monitored daily and weighed/sampled at seven day intervals starting at Day 0 before the treatment. At Day 35 of the experiment 5 pigs per group were euthanatized by intracardial injection of 0.3 mL/kg of T61 preparation (Hoechst, München, Germany) and sampled for immunohistology.

### Monoclonal antibodies (mAbs) and conjugates

The mAbs reactive with swine leukocyte surface molecules *i*.*e*. cluster of differentiation (CD) antigens that we have used to study identification/quantification patterns of respective lymphoid cell subsets are listed in Table 1.

**Table 1:** Murine mAbs specific for swine leukocyte surface CD antigens and conjugates used in cytometric immunophenotyping of peripheral blood lymphoid cell from weaned pigs

**Clone(mAb)/pAb**	**Isotype**	**mAb specificity**	**Conjugate**	**Targeted cells/molecule**	**Origin**
74-12-4	IgG2b	CD4	Pe/Cy5®	Helper T lymphocytes	Abcam, Cambridge, UK
76-2-11	IgG2a	CD8a	Phycoerythrrine	Cytolytic T lymphocytes	
K252-1E4	IgG1	CD45	FITC	Lymphoid cells	AbD Serotec, Kidlington, Oxford, UK
BB6-11C9.6	IgG1	CD21	FITC	B lymphocytes	Abcam, Cambridge, UK

The primary and secondary antibodies (Abs) that were used to study *in situ* identification, distribution and quantification patterns of CD45RA^+^ lymphoid cells residing ileal mucosa of weaned pigs are listed in Table 2.

**Table 2:** **Primary and secondary Abs used for immunohistological identification**/**localization and morphometric quantification of CD45**^+^**lymphoid cells residing ileal mucosa of 9**-**week**-**old pigs** (**N** = **2**) **following 5 weeks after the treatment with a single dose of 10 mL of POE**-**POP copolymer**

**Abs**	**Targeted marker**	**Isotype**	**Clone/conjugate**	**Cellular/molecular specificity**	**Origin**
Primary mAb	CD45RA	IgG1	MIL13	Subset of immunologically naive lymphoid cells	AbD Serotec, Kidlington, Oxford, UK
SecondaryAbs	Mouse IgG	IgG	IgG:HRP	Rabbit Ab against mouse IgG	Abcam, Cambridge, UK
	Goat IgG	IgG	IgG:HRP	Goat Ab against rabbit IgG	

### Productivity parameters

The pigs were weighed at weekly intervals during the experiment and changes in their body mass were recorded. The changes of body mass within experimental group of pigs were calculated based on differences between either body weight at the beginning of the experiment (Day 0 = 100% of body mass) or average group body weight at Day 7, 14, 21, 28 and 35 of the experiment in comparison to average body weight of the pigs from the control group. Feed intake was recorded on a weekly basis, and at the end of experiment a total group feed inake, feed conversion ratio and total group body weight gain were calculated, in relation to Day 0.

### Clinical observation

The pigs were monitored daily for diarrhea and/or the other clinical signs of health disorders, and the incidence/severity of diarrhea were recorded. Severity of diarrhea was scored as follows: 0 = normal feces, 1 = soft feces, 2 = fluid feces, 3 = projectile diarrhea. Beside morbidity, the mortality was also monitored, and dead pigs were necropsied and examined for gross pathology changes.

### Blood sampling

At the same weekly intervals the blood samples (1 ml) were collected from *vv*. *cava cranialis* into glass tubes (Beckton Dickinson, Plymouth, UK) with EDTA (Sigma) containing anticoagulant for flow cytometry analysis.

### Flow cytometry

A single cell suspensions (100 μL) were prepared in triplicates (comprising 10,000 cells each) and incubated with mAbs (50 μL) (Table 1) and processed as described previously [29]. The fluorescence of the mAb-labelled porcine lymphoid cells was quantified using a Coulter EPICS-XL flow cytometer (Beckman Coulter, Miami, FL, USA) as detailed earlier [30]. The isotype-matched mouse immunoglobulins were used to detect a nonspecific fluorescence in the control cell suspensions.

### Intestinal sampling

Immediately following euthanasia (at Day 0 and Day 35) 5 specimens of mid ileum from each of 5 pigs per group (either 5 to 6 cm or 7 to 8 cm proximal to the ileocaecal junction of 4-week-old pigs and 9-week-old pigs, respectively) were fixed in 10% neutral-buffered formalin (pH 7.0-7.6) for 24 hours until used for histopathology and immunohistology analyses.

### Histopathology and immunohistology

After fixation the specimens of ileum were dehydrated, embedded in the paraplast (Sigma, Sherwood Medical Industries, USA), cut into 5 μm thick serial sections and then processed for a standard hemalaun (Meyer’s solution; Kemika, Zagreb, Croatia) and eosin staining. These sections were examined by a light microscope (Leitz, Orthoplan, Germany) in order to identify areas suitable for immunohistological identification/quantification of the ileal CD45RA^+^ lymphoid cells. For immunohistology the paraplast-embedded sections were processed by an indirect immunoperoxidase (IP) method using primary and secondary Abs (Table 2) as detailed earlier [31]. After drying the sections were examined by a light microscope (Eclipse E600, Nikon, Japan) and the areas selected for histomorphometry were photographed by a digital camera (DMX1200, Nikon, Japan).

### Histomorphometry

Histomorphometric analysis of lymphoid cells within compartments of ileal mucosa was performed using commercial software imaging program Lucia G (version 4.11) for digital image analysis (DIA). The quantification of CD45RA^+^ cells was performed by DIA in 12 randomly selected tissue section fields at x200 on screen magnification. Such counting included interfollicular areas (IFA) and follicular areas (FA) of the ileal Peyer’s patches (PP). The results are expressed as the mean values of the number of cells per μm^2^ of an average tissue section field of 71.168,87 μm^2^. Conducted prior testing showed that when more than 12 fields in each of 5 tissue samples from one pig were analysed there were no statistically significant deviations in cell counts of individual pigs [32]. The obtained differences in the mean values of tested cells between the treatment group and the control pigs were evaluated by the statistical analysis as described below and also calculated as the index of increase or decrease of the number/percent of these cells in relation to the values obtained for the control pigs.

### Statistical analysis

Numerical data were analysed by Student’s *t* test for dependent samples, because there were only two groups of animals (i.e. the POE-POP treated group and the control group) using the StatisticaSixSigma software (StatSoft, Inc.). Significance of differences between treated and control groups of pigs were considered as significant at p < 0.05 and lower values.

## Results

Pigs treated with POE-POP had a significant increase in live body weight compared to the control pigs from Day 14 to Day 35 of the trial (Table 3).

**Table 3:** **Comparison of body weight** (**BW**) **changes in weaned pigs treated at Day 0 with POE**-**POP during 5 weeks of the experiment**

**Day of experiment**	**Mean BW(kg)of pigs treated with:**		**Difference (POE-POP *vs.*Saline)**	**p - value**
	**Saline**^**a**^	**POE-POP**^**b**^		
**0**	7.2	7.17	- 0.03	0.928
**7**	8.87	9.51	0.64	0.158
**14**	10.05	11.12	1.07*	0.024
**21**	12.05	14.23	2.18**	0.003
**28**	13.42	17.43	4.01***	0.00009
**35**	17.61	20.91	3.30*	0.0108

^a, b^Single *p*.*o*. application of 10 ml of either saline (^a^) or POE-POP (^b^) at Day 0; groups comprised 20 pigs each:Values differ significantly at *p < 0.05, **p < 0.01 or ***p < 0.001; unmarked differences were not significant.

They had higher average body weight at the end of the experiment (~ 21 kg) than control pigs (~ 18 kg), and the higher body weight gain in relation to Day 0 (191.63% *vs*. 144.58%) as well as in relation to the control pigs (for 18.74%), with a lower feed conversion ratio (for 26.73%) from that recorded in the control pigs. In order to determine the kinetics of growth in experimental pigs, feed intake and feed conversion, we have analyzed average daily gain (ADG) gain, average daily feed intake (ADFI), and feed conversion ratio (FCR) during the experimental period of 5 weeks following weaning (Table 4).

**Table 4:** **Average daily gain** (**ADG**), **average daily feed intake** (**ADFI**), **and feed conversion ratio** (**FCR**) **per week in weaned pigs treated at Day 0 with POE**-**POP after 5 weeks of the experiment**

**Treatment**^**a**^	**Parameter**	**Mean**					
		**0 - 7**	**7 - 14**	**14 - 21**	**21 - 28**	**28 - 35**	**0 - 35**
**Saline**	**ADG(g)**	214	172	273	261	562	296
	**ADFI(g)**	419	464	780	565	975	641
	**FCR**	1.96	2.70	2.86	2.16	1.74	2.17
**POE**-**POP**	**ADG(g)**	292	268	445	457	496	392
	**ADFI(g)**	402	483	680	685	868	624
	**FCR**	1.37	1.80	1.52	1.50	1.75	1.59

^a^Single *p*.*o*. application of 10 ml of either saline or POE-POP at Day 0; groups comprised 20 pigs each.

The pigs treated with POE-POP had better ADG during the first 4 weeks, with the exception of the period between Day 28 and Day 35 of the experiment, when the control pigs had slightly better growth. Hence, during the entire experimental period (from Day 0 to Day 35) the treated pigs had considerably higher ADG (392±14 g) and lower FCR (1.59 kg), but slightly lower ADFI (624 g) as compared to the values obtained for the controls (296±76 g; 2.17 kg; 641 g, respectively).

In order to evaluate gut health we have monitored the incidence and severity of diarrhea induced by intestinal infections during the experimental period. A much lower combined diarrhea severity score (DSS) was recorded in pigs treated with POE-POP (5 or ~ 91%) than in the control pigs (Table 5).

**Table 5:** **Incidence and severity of diarrhea**, **and mortality in weaned pigs treated at Day 0 with POE**-**POP after 5 weeks of the experiment**

**Treatment**^**a**^	**No.of diarrheic pigs/total no.of pigs(%)**^**b**^	**DSS**		**ADS**		**No.of dead pigs/total no.of pigs (%)**
		**Sum of DSS**^**c**^	**% difference POE-POP*vs*.Saline**	**ADS ratio**^**d**^	**% difference POE-POP*vs*.Saline**	
**Saline**	12/20 (60)	54	/	1.54	/	5/20 (25)
**POE**-**POP**	5/20 (25)	5	- 90.74	0.14	- 90.91	0/20 (0)

^a^Single *p*.*o*. application of 10 ml of either saline or POE-POP at Day 0; groups comprised 20 pigs each.

^b^During 35 days of the experiment.^c^DSS: 0 = normal feces, 1 = soft feces, 2 = fluid feces or 3 = projectile diarrhea as summarized during 35 days of the experiment. ^d^Sum of DSS/35 days.

The higher average diarrhea severity (ADS) was recorded in the control pigs (1.54), whereas the pigs that received POE-POP had much lower ADS (0.14 or ~ 91%) after 35 days of the experiment. None of the pigs treated with POE-POP died during the experimental period whereas a rather high mortality rate (25%) was recorded in the control pigs.

Recruitment of circulating immune cell subsets was assessed by the cytometric analysis of proportions/kinetics of CD45^+^ lymphoid cells (Table 6), CD4^+^ (Table 7) and CD8^+^ T cells (Table 8) as well as of CD21^+^ B cells (Table 9) in the peripheral blood of weaned pigs during 5 weeks following the treatment.

**Table 6:** **Difference in proportion of CD45**^+^**limphoid cells in the peripheral blood of weaned pigs treated at Day 0 with POE**-**POP after 5 weeks of the experiment**

**Treatment**^**a**^	**% of CD45**^**+**^**cells in the peripheral blood (Mean values)**^**b**^**/day of the experiment**					
	**0**	**7**	**14**	**21**	**28**	**35**
**Saline**	55.31	56.28	58.30	60.51	61.27	61.46
**POE**-**POP**	49.90	52.23	59.76	66.09	72.41	76.37
**Difference** (POE-POP *vs*. Saline)	- 5.41*	- 4.05**	1.46	5.58*	11.14*	14.91*

^a^Single *p*.*o*. application of 10 ml of either saline or POE-POP at Day 0; groups comprised 7 pigs each. ^b^Values differ significantly at *p < 0.01 or **p < 0.05; unmarked differences were not significant.

**Table 7:** **Difference in proportion of CD4**^+^**T cells in the peripheral blood of weaned pigs treated at Day 0 with POE**-**POP after 5 weeks of the experiment**

**Treatment**^**a**^	**% of CD4**^**+**^**cells in the peripheral blood (Mean values)**^**b**^**/day of the experiment**					
	**0**	**7**	**14**	**21**	**28**	**35**
**Saline**	19.34	19.70	20.46	21.18	21,53	21.60
**POE**-**POP**	17.45	18.65	21.34	23.13	25.34	25.29
**Difference** (POE-POP *vs*. Saline)	- 1.89*	- 1.05	0.88	1.95*	3.81**	3.69**

^a^Single *p*.*o*. application of 10 ml of either saline or POE-POP at Day 0; groups comprised 7 pigs each. ^b^Values differ significantly at *p < 0.05 or **p < 0.01; unmarked differences were not significant.

**Table 8:** **Difference in proportion of CD8**^+^**T cells in the peripheral blood of weaned pigs treated at Day 0 with POE**-**POP after 5 weeks of the experiment**

**Treatment**^**a**^	**% of CD8**^**+**^**cells in the peripheral blood (Mean values)**^**b**^**/day of the experiment**					
	**0**	**7**	**14**	**21**	**28**	**35**
**Saline**	11.06	11.14	11.73	12.10	12.27	12.34
**POE**-**POP**	9.98	10.45	11.95	13.22	14.48	14.44
**Difference** (POE-POP *vs*. Saline)	- 1.08*	- 0.69	0.22	1.12**	2.21*	2.10**

^a^Single p.o. application of 10 ml of either saline or POE-POP at Day 0; groups comprised 7 pigs each. ^b^Values differ significantly at *p < 0.01 or **p < 0.05; unmarked differences were not significant.

**Table 9:** **Difference in proportion of CD21**^+^**B cells in the peripheral blood of weaned pigs treated at Day 0 with POE**-**POP after 5 weeks of the experiment**

**Treatment**^**a**^	**% of CD21**^**+**^**cells in the peripheral blood (Mean values)**^**b**^**/day of the experiment**					
	**0**	**7**	**14**	**21**	**28**	**35**
**Saline**	22.07	22.11	23.16	24.35	24.51	24.52
**POE**-**POP**	19.89	20.89	23.90	26.43	28.97	29.04
**Difference** (POE-POP *vs*. Saline)	- 2.18*	- 1.22	0.74	2.08	4.46*	4.52**

^a^Single *p*.*o*. application of 10 ml of either saline or POE-POP at Day 0; groups comprised 7 pigs each. ^b^Values differ significantly at *p < 0.05 or **p < 0.01; unmarked differences were not significant.

The proportion of CD45^+^ cells in the pigs that were treated with POE-POP was much higher than in the control pigs during the last three weeks of the experiment. During that period (from Day 21 to Day 35) we have recorded significantly increased proportion of CD45^+^ lymphoid cells (p < 0.01). Conversely, the proportion of these cells was significantly lower in POE-POP-treated pigs at Day 0 (p < 0.01) and Day 7 (p < 0.05) of the experiment (Table 6).

The pigs that were treated with POE-POP had an increased proportion of CD4^+^ cells (at p < 0.05, p < 0.01 or p < 0.01, respectively) and CD8^+^ cells (at p < 0.05, p < 0.01 or p < 0.05, respectively) at Day 21, Day 28 and Day 35 of the experiment as well as of CD21^+^cells at Day 28 and 35 of the experiment (at p < 0.05 or p < 0.01, respectively) (Tables 7, 8 and 9).

Interestingly, these pigs had a significantly lower proportion of T and B subsets tested (at p < 0. 05 or p < 0.01) than those recorded in the control pigs at Day 0 of the experiment (Tables 7, 8 and 9).

**Figure 1:**
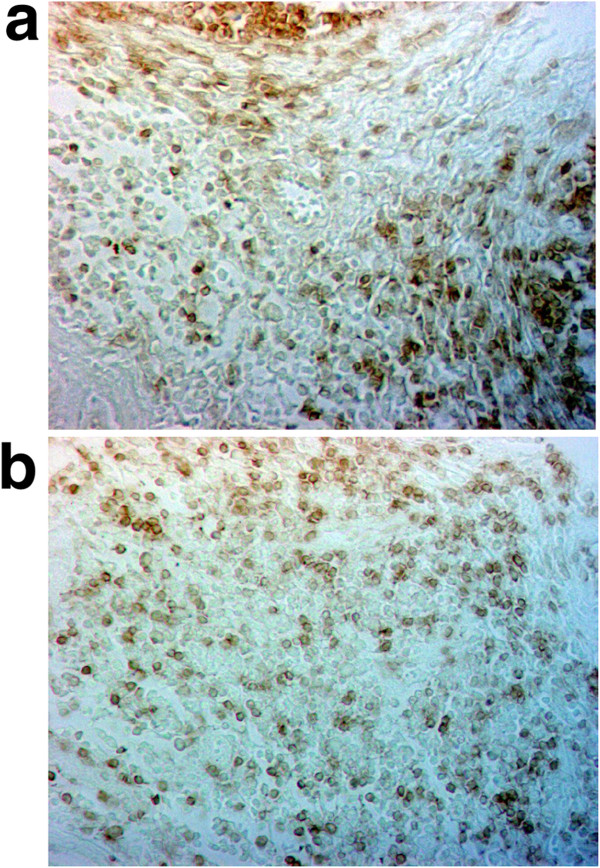
**CD45RA**^**+**^**lymphoid cells in the IFA of ileal PP of the pig from control group (a) and of the pig treated with POE-POP (b) after 5 weeks of the experiment; an indirect IP method, x 400 magnification.**

**Figure 2:**
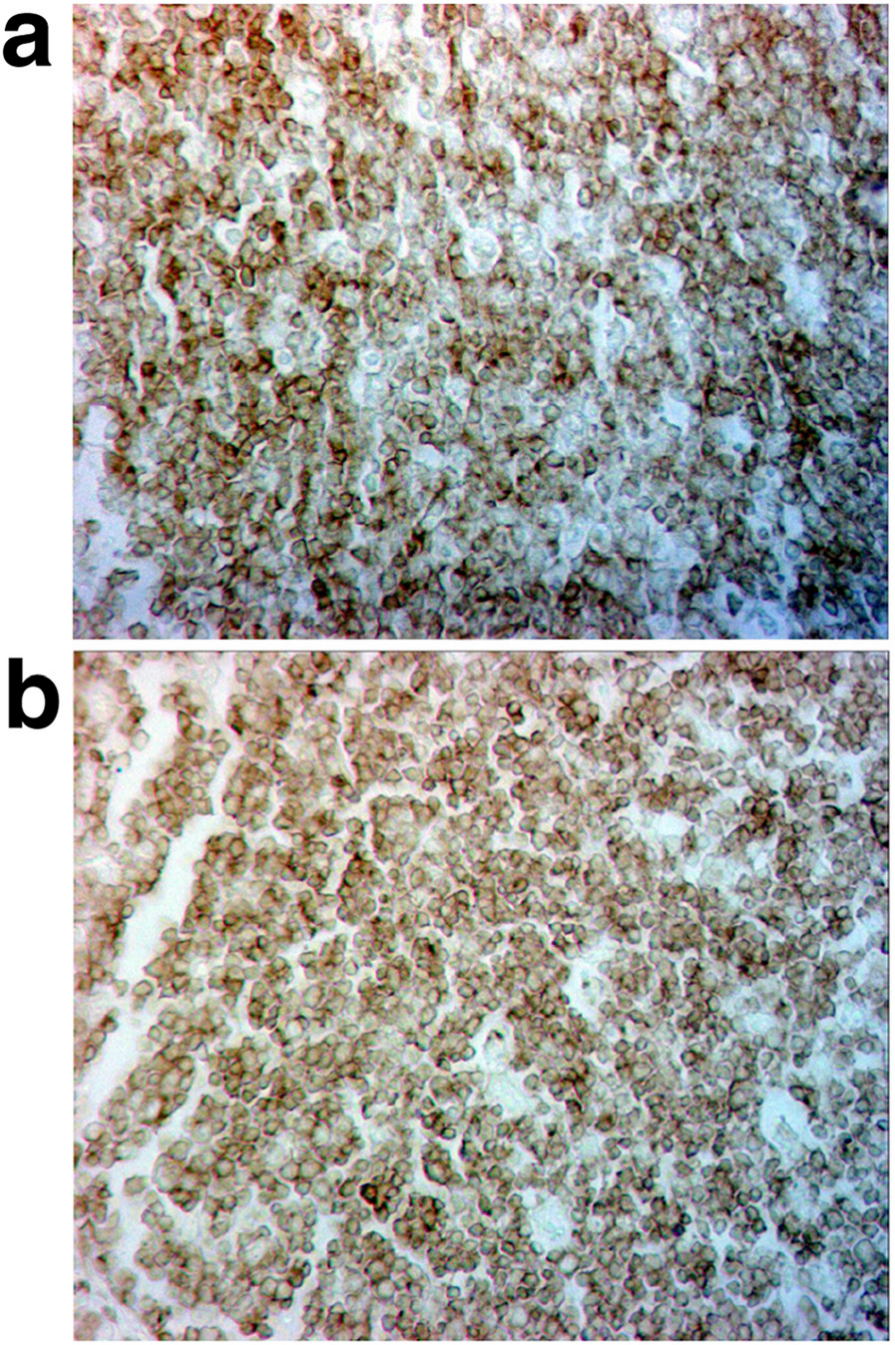
**CD45RA**^**+**^**lymphoid cells in the FA of ileal PP of the pig from control group (a) and of the treated with POE-POP (b) after 5 weeks of the experiment; an indirect IP method, x 400 magnification.**

Distribution patterns of CD45RA^+^ naïve lymphoid cells within IFA and FA of the ileal PP from the POE-POP-treated pigs after 5 weeks of the experiment are shown in Figures 1 and 2, respectively.

Numerous CD45RA^+^ cells were seen in the lamina propria of intestinal villi, within Lieberkühn’s crypts and in the submucosa. Small numbers of these cells were found adjacent to the basal membrane of epithelial cells. Distribution of greater number of CD45RA^+^ naive lymphoid cells was demonstrated in the middle of the villous lamina propria and in the IFA (Figure 1a, b). A much greater frequency of these cells was observed in the FA of the ileal PP (Figure 2a, b). As can be assessed by microscopic examination it seems that CD45RA^+^ cells were less numerous within both lymphatic compartments tested (IFA and FA of the ileal PP) in 4 week-old pigs (at Day 0) than in 9 week-old pigs (Day 35) (Figures 1 and 2).

Such assumption was confirmed by the histomorphometric analyses of these cells within both IFA and FA of the ileal PP of weaned pigs following 5 weeks of the treatment (Table 10).

**Table 10:** **Numbers of CD45RA**^+^**naïve lymphoid cells in IFA and FA of the ileal PP of pigs treated at Day 0 with POE**-**POP after 5 weeks of the experiment**

**Treatment**^**a**^	**Mean no.of CD45RA**^**+**^**cells**^**b**^**in**		**Index of increase/decrease and % of increase of the mean no. ileal CD45RA**^**+**^**cells in**^**c**^	
	**IFA**	**FA**	**IFA**	**FA**
**Saline**	85.80	504.80	1,00	1,00
**POE**-**POP**	173.60	784.00	+1.76	+1.55
**Difference** (POE-POP *vs*. Saline)	87.80*	279.20**	76%	55%

^a^Single *p*.*o*. application of 10 ml of either saline or POE-POP at Day 0; 5 pigs per group were euthanatized and sampled at Day 35 of the experiment. ^b^No. of the cells per μm^2^ of randomly selected 12 microscopic fields (average area of digital image field was 71.168,87 μm^2^) in each of 5 serial sections of mid ileum per pig. ^c^Increase (+) or decrease (−) of the mean values in treated pigs vs. the mean values in control pigs (1.0 or 100%). Values differ significantly at *p < 0.05 or ** p < 0.01.

We have recorded that stimulation of pigs with POE-POP significantly increased the number of their CD45RA^+^ cells in IFA (p < 0.05) and FA (p < 0.01) of the ileal PP as compared to the values obtained in the control pigs.

## Discussion

Based on these results we have concluded that the copolymer of POE-POP have shown to be effective as an immunomodulator in stimulating non-specific recruitment of circulating and intestinal immune cell subsets which resulted in the reduction of incidence/severity of diarrheal disease and maintenance of gut health of the waenlings, and thus could be of importance in promoting the resistance of weaned pigs to enteric infections.

Significant changes occurred in the proportion of peripheral blood CD45^+^, CD4^+^, CD8^+^ and CD21^+^cell subsets from either Day 21 to Day 35 or from Day 28 to Day 35 of the experiment, respectively, as well as in the number of ileal CD45RA^+^ lymphoid cells at Day 35 following the treatment with POE-POP (at Day 0 or at weaning). The role of these cells could be to promote more robust, rapid and sustainable protection of weaned pigs from antigenic challenges since protection from passive immunity declines and ceases within the first two weeks after weaning. Furthermore, the copolymer positively influenced feed intake which resulted in: (i) a higher average body weight of principal *vs*. control pigs at the end of the experiment (Day 35), (ii) higher body weight gain of principals in the relation to Day 0 as well as in the relation to the controls, and (iii) a significantly lower feed conversion ratio in the principals than in the controls. These favorable effects of the copolymers on both immunity and productivity of weanlings seem to be interactively connected by improved gut health and physiology.

Although the influence of POE-POP on growth and performance has been described earlier [22], to date, such approach was not performed in domestic food animals, including swine. Also, as we did not find any data on modulating porcine immunity by the IRM-active block copolymers, we attempted to explain our results by relating data on their molecular conformation and physico-chemical properties to those of hormones (such as somatostatin and ACTH) and neuropeptides (β-endorphin) which may produce similar effects [4]. Specifically, it is well known that porcine neuroendocrine and immune systems are interactively communicating by means of these molecules [33]. Accordingly, it is logical that POE-POP may stimulate, depending on dose applied and time of the exposure, agonistic and antagonistic effects of the aforementioned hormones and neuropeptides [25]. The effectiveness of POE-POP to control incidence and severity of diarrheal disease was expressed as the sum of DSS and was found to be much lower (for 90.74%) in the treated than in the control pigs (5 *vs*. 54). A much higher ADS ratio was recorded in the control pigs (1.54 *vs*. 0.14), whereas the pigs that received POE-POP had much lower ADS (for 90.91%) after 35 days of the experiment. These effects of the coploymers could be ascribed to their ability to facilitate the transport of ions through the cell membrane [25], their microbicidity in the gut and mediation of opsonization and complement activation [8] as well as to their adherence to lipids providing capture of protein antigens within the local tissue [10] and facilitation of antigen uptake by the APC [13].

Regarding the imunomodulatory properties of POE-POP it has been reported that the copolymer stimulated both humoral and cellular immunity [5,6], particularly production and maturation of T cells in thymus as well as proliferation of the immune cells in lymph nodes and bone marrow [25,30]. These data agree well with our finding that POE-POP may enhance the proliferation of both circulating and residential CD45^+^ and CD45RA^+^, respectively, in weaned pigs. Our previous findings of increased proliferation of CD8^+^ cytolytic T cells in the jejunal lamina propria, CD4^+^ helper T cells in the ileal PP and SWC5^+^ γδ TCR^+^ null cells (probably NK-cells) in the mesenteric lymph node (MLN) [17], when POE-POP was applied as adjuvant in the combination with the vaccine candidate porcine non-ETEC strain, also support results of the current study in which we have demonstrated increased number of ileal CD45RA^+^ naïve lymphoid cells. Interestingly, the copolymers differently affected CD1^+^ and CD21^+^ B cell subsets, *i*. *e*. induced either their increase in PP and MLN or their decrease in MLN and PP, respectively [17]. Such immunostimulatory effects of the copolymers on Th1 and Th2 responses following the facilitation of uptake of exogenous antigens by APC have been reported [13]. The diversity of the immune cell subsets that could be stimulated by POE-POP in the postweanling pig model system, it suggests that the copolymers when applied as the adjuvant with specific vaccinal immunogen (non-ETEC strain) or alone as an IRM (applied in the current study), may affect either the immunologically commited (T, B and NK-cells) or naïve (CD45RA^+^) lymphoid cells [29]. Consistent with a former finding of selectively activated CD1^+^ and CD21^+^ gut-residing B cells is our previous finding of stimulated proliferation of IgA^+^ plasma cells and sIgA antibodies by the immunization with POE-POP adjuvanted non-ETEC vaccine candidate strain [20,32]. Also, more recent finding that the copolymer increased proportion of peripheral blood lymphocytes in weaned pigs [26] is in accordance with the highly elevated T (CD4^+^, CD8^+^) and B (CD21^+^ ) cells observed in this study. As we did not find similar data for pigs we may only quote those found in rodents which suggesting that POE-POP stimulated antibody production, including mucosal antibody responses [10]. However, depending on the quantity of POE in the copolymers they may also stimulate cellular immunity [14]. Recently, the copolymers synthesized using various amonts of POE and POP and with different arrangements of their blocks elicited different immune responses, which were always Th1 specific in mice [21].

The health problems in swine, particularly young pigs, kept under intensive conditions of rearing cause significant economic losses worldwide.As a consequence of the European ban of in-feed antibiotic growth promotors (AGP), new strategies that include natural and/or synthetic IRMs were developed to increase the resistance to diseases in food animals. In swine production, this is of particular importance during the weaning transition. Strategies aimed at stimulating both innate and acquired immunity in weaned pigs through the use of non-AGP substances able to modulate their immune functions have gained increasing interest in veterinary immunology [34].

## Conclusions

The property of perorally given POE-POP to stimulate both systemic and intestinal cellular immunity in weaned pigs may allow the use of these active block copolymers as the IRMs, particularly when targeted to the GALT well known to promote rather tolerogenic than protective immune responses.

## Competing interests

The authors declare that they have no competing interests.

## Authors’ contributions

HV, IV, GM and MP designed the study protocol. DM, DC, DK and DŠ performed the clinical study procedures. HV, GL, BG, DA and KV prepared and analyzed the samples. HV and MMP performed pharmacological analyses. AP performed immunohistological analyses. SS revised statistical analyses and reinterpreted obtained significant differences. All authors contributed and approved the final manuscript.
